# A Review of Thrombopoietin Receptor Agonists and Subsequent Development of Thrombosis: Exploring a Hematologic Paradox

**DOI:** 10.7759/cureus.115715

**Published:** 2026-09-03

**Authors:** Gillian Graifman, Ranya Parekh, Rajesh Varma C P, Arun Nair

**Affiliations:** 1 Pediatrics, Cohen Children's Medical Center, New Hyde Park, USA; 2 Medicine, Hofstra University/Donald and Barbara Zucker School of Medicine, Hempstead, USA; 3 Pediatrics, Saint Peter's University Hospital, New Brunswick, USA; 4 Pediatric Hematology Oncology, Cohen Children's Medical Center, New Hyde Park, USA

**Keywords:** hemostasis, platelets, thrombocytosis, thrombopoietin receptor agonists, thrombosis

## Abstract

Thrombopoietin receptor agonists (TPO-RAs) are drugs that increase platelet production and reduce bleeding risk. Agents such as romiplostim, eltrombopag, avatrombopag, and lusutrombopag act in different ways to stimulate megakaryopoiesis. These agents are used in both adult and pediatric populations, primarily in chronic immune thrombocytopenia, severe aplastic anemia, and myelodysplastic syndrome, as well as pre-procedural in patients with chronic liver disease. This literature review summarizes the current TPO-RAs and aims to evaluate the risk of thrombus formation. Past studies have been inconsistent in significantly associating thrombotic events with higher platelet counts, medication doses, or drug mechanisms of action. The pathophysiology appears to be multifactorial, with some theories suggesting risk from increased thrombopoiesis with potential alterations in platelet activity or an already prothrombotic patient state due to underlying disease or individual predisposing factors. Risks such as prior thrombus formation, older age, history of splenectomy, antiphospholipid syndrome, prolonged TPO-RA exposure, and thrombotic risk associated with the underlying disease being treated are important to consider not only during treatment initiation but also during continued risk assessment.

## Introduction and background

Thrombopoietin receptor agonists (TPO-RAs) are a class of medications used primarily to stimulate megakaryopoiesis, the production and maturation of platelets in the bone marrow [1]. Examples of TPO-RAs currently in use include romiplostim, eltrombopag, avatrombopag, and lusutrombopag [2,3], which will be the primary focus of this literature review.

Thrombopoietin (TPO) is an endogenous hormone primarily made in the liver, produced to increase thrombopoiesis, or platelet production [4]. Receptor binding activates Janus kinase/signal transducers and activators (JAK/STAT) and mitogen-activated protein (MAP) kinase pathways, promoting the growth, differentiation, and maturation of platelet-producing cells. The body uses a negative feedback loop to maintain a normal platelet range, increasing TPO production when platelet count is low, and vice versa [5]. TPO-RAs, also known as TPO mimetics, mimic the body’s natural TPO by binding to megakaryocyte receptors, namely c-Mpl, in the bone marrow [2], with the goal of ultimately increasing platelets. They are most useful in treating immune thrombocytopenia (ITP), myelodysplastic syndromes (MDS), aplastic anemia, and chemotherapy-induced thrombocytopenia, and in preparation for invasive procedures [2].

ITP is an autoimmune disorder characterized by an isolated platelet count less than 100x10^9^/L, most commonly presenting with bruising, petechiae, or epistaxis [6]. Treatments include observation, corticosteroids, platelet transfusions, intravenous immunoglobulin (IVIG), rituximab, and splenectomies [7], with TPO-RAs often used as second-line treatments [8]. The most widely known TPO-RAs, romiplostim and eltrombopag, were FDA-approved in 2008 for treatment of refractory and chronic ITP in adults. This expanded to include children aged one year and older, with eltrombopag approved in 2016 and romiplostim in 2018 [9].

In MDS, a disease in which the bone marrow produces defective blood cells, TPO-RAs, specifically eltrombopag, have been used for supportive care. While disease-modifying drugs treat MDS, eltrombopag reduces the need for platelet transfusions [10]. Concerns have historically been raised regarding the potential for TPO-RAs to increase marrow blasts or promote progression to acute myeloid leukemia in patients with MDS [2]. However, more recent pooled randomized evidence has not demonstrated a statistically significant increase in AML transformation with TPO-RA therapy compared with control groups (RR 1.16; 95% CI, 0.88-1.52) [10].

In aplastic anemia, the bone marrow is essentially “empty” and does not produce blood cells. A 2017 NEJM study showed that adding eltrombopag to standard immunosuppression with antithymocyte globulin and cyclosporine improved cell production [11], and current guidelines recommend adding eltrombopag in adults and children with severe aplastic anemia [12].

The discussion of TPO-RA use is not complete without an in-depth investigation of its side effects. Although the goal of TPO-RAs is to increase platelet synthesis through multiple different pathways, one side effect of interest that has been investigated is the risk of thrombus formation. A study conducted by Zordan et al. in 2026 suggested that in adults with ITP, there was a significantly increased risk of thrombotic events over one year when using eltrombopag (5.1%) compared to romiplostim (1.9%) [13]. Conversely, a systematic review and meta-analysis conducted by Shen et al. in 2024 [14] showed no significant differences in thrombosis risks between different TPO-RAs overall. It did, however, show an increased risk within the ITP treatment group. This leads us to the question about the risk of thrombosis with different types of TPO-RAs, and, if there is a statistically significant risk, what measures should be used to screen, prevent, and modify treatment accordingly.

This literature review aims to examine the available TPO-RAs that are being used in both the pediatric and adult populations and to examine the risk of thrombosis formation and subsequent screening and preventive methods suggested in the literature and guidelines. The goal of this study is to provide a comprehensive overview and suggest potential guideline modifications pertaining to the use of TPO-RAs in both populations.

## Review

Methods

The study is designed as a narrative literature review. The team carefully discussed, analyzed, and agreed on the research questions before further developing the study. Articles were curated using PubMed and Google Scholar with the following inclusion criteria: English-language articles, human studies, and studies relevant to TPO-RAs and investigation into subsequent thrombosis formation. Keywords used to curate articles included "Thrombopoietin Receptor Agonists", "Thrombus Formation", "Immune Thrombocytopenia," and "Aplastic Anemia". Because this study was a narrative rather than a systematic literature review, we did not apply a predefined search period, a formal record-level screening process, or a standardized risk-of-bias assessment. The keywords were combined using Boolean operators (AND/OR) to retrieve the most relevant articles. Once articles were sourced, team members were assigned sections of the literature review for analysis and drafting. Other team members then cross-analyzed these sections to ensure the validity of the information, a consistent writing style, and corrections when necessary. The final manuscript was approved after this editorial process and reviewed by two members of the study team before submission.

Pathophysiology of thrombosis formation with TPO mimetics

Platelets are an integral part of hemostasis necessary to form clots and stop bleeding. However, when stasis of blood occurs, it propagates thrombus formation, leading to obstruction of flow through the vessel, as described in Virchow's triad [15]. Virchow's triad outlines three scenarios that can increase thrombus formation: endothelial injury, stasis of blood, and a hypercoagulable state [16]. While endogenous TPO is not directly responsible for the formation of thrombi, it plays a role through increased platelet production. Thus, when either endogenous TPO or TPO-RA levels are high, thrombocytosis (elevated platelets) allows for available platelets to form clots and create a hypercoagulable state.

Table 1 is an overview of the current TPO-RAs available in the market and their unique qualities.

**Table 1:** Available TPO-RAs Comparison of available TPO-RAs, including mechanism of action, route of administration, half-life, and FDA-approved indications [2,17-24]. TPO-RAs: thrombopoietin receptor agonists; ITP: immune thrombocytopenia

Drug	Mechanism	Route of Administration	Half-Life	FDA-Approved Indications
Eltrombopag	Activates transmembrane portion of TPO-R	Oral (empty stomach)	21–32 hours	Severe aplastic anemia, refractory/chronic ITP, thrombocytopenia in chronic hepatitis C
Romiplostim	Activates extracellular domain of TPO-R	Subcutaneous weekly	1–34 days	ITP
Avatrombopag	Activates transmembrane portion of TPO-R	Oral (with food)	19 hours	Chronic liver disease, chronic ITP, persistent ITP in pediatric patients
Lusutrombopag	Activates transmembrane portion of TPO-R	Oral (with or without food)	27 hours	Adults with chronic liver disease require it to be used prior to a procedure

Eltrombopag works by stimulating megakaryocyte precursor cells to promote differentiation by binding to a receptor site distinct from endogenous TPO [2]. It is best known for its use in combination with immunosuppressive therapy as first-line treatment for severe aplastic anemia in adults and children >2 years of age [17,18].

Romiplostim is a peptide that extracellularly binds to the same receptor as naturally occurring TPO, stimulating mature precursor cells to increase platelet production [2,19,20]. It is primarily used for adults and children >1 year of age after first-line treatment failure of chronic ITP. Unlike other TPO-RAs, it is not approved for MDS or other conditions causing low platelets [21].

Avatrombopag and lusutrombopag work similarly to increase platelet production. They are FDA-approved for patients with chronic liver disease (CLD) undergoing invasive procedures [9,22]. Avatrombopag was approved in 2019 and has a unique mechanism of action in that it increases platelet production by selectively binding to the TPO receptor without competing with endogenous TPO. It stimulates cell growth in a dose-dependent manner and is therefore receptor-dependent [23]. Lusutrombopag is an oral small-molecule TPO-receptor agonist that activates c-Mpl signaling, stimulating megakaryocyte proliferation and differentiation, and thereby increasing endogenous platelet production. Its mechanism is essentially similar to avatrombopag, with the major differences being dosing and administration [24]. Lusutrombopag has a fixed dose and can be taken with or without food, whereas avatrombopag requires dose escalation to achieve the desired platelet count and must be taken with food [24].

Despite the differences in receptor binding and pharmacologic properties among the TPO-RAs, the pre-existing consensus is that there is insufficient evidence to suggest that a particular mechanism of TPO-receptor activation is inherently more thrombogenic. All the agents ultimately stimulate megakaryopoiesis and increase endogenous platelet production, raising the question of whether the increased risk of thrombosis reported with TPO-RA therapy is simply a consequence of an increased circulating platelet count. However, available evidence suggests that the relationship between TPO-RAs and thrombosis is considerably more complex [2].

An intuitive mechanism for TPO-RA-associated thrombosis is that increased thrombopoiesis results in a larger circulating platelet mass available to participate in thrombus formation. However, thrombotic events during TPO-RA therapy have not demonstrated a consistent relationship with the degree of thrombocytosis. In clinical studies, thromboembolic events have occurred across a wide range of platelet counts, with approximately 30-50% of reported events occurring when platelet counts were below the normal range [2]. Furthermore, thrombotic events have not consistently been associated with either thrombocytosis or higher doses of TPO-RAs [2]. These observations suggest that an increased platelet count alone is insufficient to explain the thrombotic events observed with these agents.

Another potential explanation is that stimulation of megakaryopoiesis could alter the characteristics of the circulating platelets. Increased platelet production results in the release of newly generated platelets, raising the possibility that changes in platelet activation or reactivity may contribute to thrombus formation, independently of the absolute platelet count. However, available evidence does not demonstrate that TPO-RA therapy produces hyperreactivity. A study by Psaila et al. evaluated platelet function in patients with ITP before and during treatment with eltrombopag and found that although patients with ITP demonstrated increased platelet activation at baseline compared with healthy controls, eltrombopag treatment did not result in significant additional platelet activation or hyperreactivity despite increasing platelet counts [25]. This suggests that the potential thrombotic risk associated with TPO-RAs cannot be explained solely by increased platelet numbers or direct enhancement of platelet reactivity.

The underlying disease may therefore play an important role in determining thrombotic risk. This is particularly relevant in ITP, which, despite being characterized by thrombocytopenia and an increased risk of bleeding, is associated with thrombotic complications [26]. Patients with ITP receiving TPO-RAs may additionally have other acquired or pre-existing prothrombotic factors, including advanced age, cardiovascular disease, prior thromboembolism, splenectomy, antiphospholipid antibodies, malignancy, immobility, and estrogen exposure [2]. TPO-RA therapy may therefore act as an additional contributor within an already prothrombotic environment rather than functioning as an independent cause of thrombosis.

Importantly, the differences in receptor binding among the individual TPO-RAs have not established a clear mechanistic hierarchy of thrombotic risk. Romiplostim interacts with the extracellular domain of the TPO receptor, whereas eltrombopag, avatrombopag, and lusutrombopag interact with the transmembrane domain; however, there is currently insufficient evidence to conclude that either mode of receptor activation is inherently associated with a greater risk of thrombosis [2]. A systematic review and meta-analysis by Shen et al. similarly did not demonstrate a statistically significant increase in thrombotic events among patients with ITP related to TPO-RAs compared with control groups [14]. Conversely, more recent observational evidence suggests that thrombotic risk may not necessarily be identical among individual agents. Zordan et al. reported a higher one-year incidence of thrombosis among adults with primary ITP treated with eltrombopag compared with romiplostim [13]. Although these findings raise the possibility of differences in thrombotic risk among individual TPO-RAs, they do not establish that differences in receptor binding or mechanism of action are responsible.

Overall, the pathophysiology of TPO-RA thrombosis appears to be multifactorial rather than simply a consequence of elevated platelet count. Increased thrombopoiesis, potential alterations in circulating platelets, underlying prothrombotic characteristics of disease, and individual risk factors may collectively contribute to thrombus formation. Consequently, understanding patient-specific risk factors is particularly important when evaluating the thrombotic risk associated with TPO-RA therapy.

Patient-specific risk factors for thrombosis while on TPO-RAs

Given the multifactorial nature of thrombosis associated with TPO-RAs, identifying pre-existing risk factors is important when assessing the safety of initiating and continuing these agents. Patients receiving TPO-RAs may have underlying disease or characteristics that independently predispose them to thrombosis. These confounders make it difficult to distinguish the medication contribution from the baseline thrombotic risks in each patient [2,26].

A history of thrombosis is important to consider when initiating TPO-RA therapy. In a systematic review and meta-analysis evaluating thrombotic events among patients with ITP receiving thrombopoietic agents, Dong et al. found a higher rate of thrombosis in studies that included patients with a history of thrombosis compared with studies that excluded these patients (3.0% vs. 1.4%, p = 0.257) [27]. Although not statistically significant, the findings suggest that patients with previous thrombosis may require greater consideration during therapy. This goes along with Ghanima et al. discussing the importance of reviewing thromboembolic history when assessing risks prior to TPO-RA initiation [2].

Advanced age may also influence thrombotic risk. While not statistically significant, Dong et al. reported a higher incidence of overall thrombotic events among patients older than age 50 compared with those younger than age 50 (3.7% vs. 1.3%, p = 0.132) [27]. More recently, Goncalves et al. studied a retrospective cohort of adults with ITP and identified older age as a significant risk factor for thrombosis, emphasizing the importance of ongoing risk assessment throughout the disease course [26].

Splenectomy represents another important consideration, particularly among patients with chronic or refractory ITP. Previous splenectomy has been associated with an increased risk of venous thromboembolism in those with ITP [28]. Similarly, the presence of antiphospholipid antibodies should be considered because these patients may have an additional underlying predisposition to thrombosis despite the presence of thrombocytopenia [29].

The underlying diagnosis itself is also relevant when identifying thrombotic risks. ITP, despite being characterized by thrombocytopenia and bleeding, is associated with an increased risk of thrombosis [26]. In a retrospective cohort of 220 adults with ITP, Goncalves et al. identified 37 thrombotic events among 26 patients during 1,365 person-years of follow-up, corresponding to an overall thrombosis incidence of 2.71 per 100 person-years [26]. Importantly, TPO-RA therapy was independently associated with thrombosis in multivariable analysis, supporting the need for continued thrombotic risk assessment in patients receiving these agents [26].

Duration of therapy may additionally influence risk, as Dong et al. found that the incidence of overall thrombotic events was significantly higher among patients receiving thrombopoietic agents for more than six months compared with those treated for less than six months (4.7% vs. 0.1%, p < 0.001) [27]. This observation is particularly relevant when TPO-RAs are used as long-term therapy in chronic ITP and again suggests that thrombotic risk assessment should continue throughout treatment rather than being limited to the time of initiation.

Taken together, available evidence suggests that thrombotic risk during TPO-RA therapy should not be assessed solely according to the platelet count achieved during treatment. Prior thrombosis, advanced age, splenectomy, antiphospholipid antibodies, the underlying thrombotic risk associated with ITP, and prolonged exposure to thrombopoietic therapy should all be considered when evaluating an individual patient’s risk [26-29]. Recognition of these factors may help identify patients who warrant closer monitoring and more careful consideration of the risks and benefits of continued TPO-RA therapy.

Current evidence of thrombosis formation

With individual studies highlighting thrombotic events with each type of TPO-RA and subsequent outcomes, it is evident that a comparative analysis on a larger scale is required to truly understand and assess the risk of thrombosis with TPO-RAs. Table 2 describes the current clinical evidence of thrombosis associated with TPO-RAs, based on the most available and recent literature.

**Table 2:** Current Clinical Evidence of Thrombosis Associated With TPO-RAs Summary of clinical studies evaluating thrombosis associated with TPO-RAs [13,14,26,27,30-35]. TPO-RAs: thrombopoietin receptor agonists; ITP: immune thrombocytopenia; RCTs: randomized controlled trials; rhTPO: recombinant human thrombopoietin

Study	Study Design/Population	Agent	Exposure/Sample	Thrombotic Findings	Key Interpretation
Kuter et al., 2013 [30]	Long-term, open-label extension; adults with chronic ITP	Romiplostim	292 patients; up to 5 years; 614 patient-years	Thrombotic events occurred in 6.5% of patients	Thrombotic events were not associated with platelet count, suggesting that platelet count alone does not explain thrombotic risk
Wong et al., 2017 (EXTEND) [31]	Long-term extension; persistent/chronic ITP	Eltrombopag	302 patients; treatment up to approximately 8 years	19 patients experienced 24 thromboembolic events; incidence 2.69 events/100 patient-years	Both arterial and venous thrombotic events occurred during long-term exposure
Jurczak et al., 2018 [32]	Phase III randomized, placebo-controlled trial; chronic ITP	Avatrombopag	49 randomized; 32 vatrombopag, 17 placebo	3 Avatrombopag-treated patients experienced thromboembolic events during the core study; an additional jugular vein thrombosis occurred during the extension phase	Events occurred across platelet counts of 39–271 x 10^9^/L, and 3 of the 4 affected patients had multiple underlying thrombotic risk factors
Peck-Radosavljevic et al., 2019 (L-PLUS 2) [33]	Phase III randomized, placebo-controlled trial; chronic liver disease with thrombocytopenia before an invasive procedure	Lusutrombopag	215 randomized; 108 lusutrombopag, 107 placebo	4 thrombotic events; 2 with lusutrombopag and 2 with placebo	Thrombotic events occurred in both treatment groups despite prospective imaging, complicating attribution of these events specifically to lusutrombopag
Tjepkema et al., 2022 [34]	Systematic review and meta-analysis; ITP	Romiplostim, eltrombopag, avatrombopag	Randomized trial evidence	Pooled RR for thrombosis 1.82 (95% CI, 0.78–4.24)	Numerical increase in thrombosis with TPO-RAs that did not reach statistical significance
Dong et al., 2023 [27]	Systematic review and meta-analysis; ITP	Romiplostim, eltrombopag, avatrombopag, rhTPO	17 RCTs; 2105 patients in analysis of overall thrombotic events	Thrombosis occurred in 33/1425 thrombopoietic-agent-treated patients versus 4/680 controls; RR 1.73 (95% CI, 0.88–3.39)	Overall RCT risk was not statistically significant; reported event rates were higher with treatment more than 6 months than less than 6 months (4.7% versus 0.1%)
Shen et al., 2024 [14]	Systematic review and meta-analysis of RCTs; ITP	Romiplostim, eltrombopag, avatrombopag, hetrombopag	1698 patients	26 thromboembolic events with TPO-RAs versus 4 among controls; OR 1.76 (95% CI, 0.78–4.00)	No statistically significant increase in overall thrombotic events was demonstrated in the randomized evidence
Goncalves et al., 2024 [26]	Retrospective real-world cohort; adults with ITP	Eltrombopag, romiplostim	220 patients; 1365 person-years	37 thrombotic events occurred in 26 patients; incidence 2.71 events/100 person-years	Both arterial and venous thrombosis occurred, and TPO-RA therapy was associated with thrombosis in the cohort
Dai et al., 2025 [35]	Systematic review and meta-analysis integrating RCTs and prospective studies; adult primary ITP	Romiplostim, eltrombopag, avatrombopag, hetrombopag	12 RCTs (1530 patients) and 11 prospective studies (1820 patients)	RCT thromboembolic events: 0.72% with TPO-RAs versus 0.23% with placebo; Peto OR 3.25 (95% CI, 1.11 - 9.51)	Unlike several earlier analyses, this analysis identified a statistically significant thromboembolic association; prospective evidence also suggested increasing arterial risk with prolonged exposure
Zordan et al., 2026 (FAITH) [13]	Target-trial emulation using a real-world cohort of adults with primary ITP	Eltrombopag versus romiplostim	2761 eltrombopag; 949 romiplostim	One-year weighted cumulative incidence of thrombosis: 5.1% versus 1.9%; adjusted HR 1.93 (95% CI, 1.11–3.34)	Suggests a possible difference between agents, although observational evidence cannot establish an intrinsic pharmacologic difference in thrombogenicity

Clinical evidence regarding thrombosis during TPO-RA therapy has accumulated from randomized trials, long-term extension studies, meta-analyses, and real-world cohorts. Long-term studies of both romiplostim and eltrombopag demonstrate that thrombotic events continue to occur during prolonged treatment [30,31]. Importantly, the long-term romiplostim study reported thrombotic events in 6.5% of patients without demonstrating an association between thrombosis and platelet count, suggesting that treatment-induced thrombocytosis alone cannot explain the observed risk [30].

Evidence from the newer oral TPO-RAs provides a similar but less extensive signal. In the phase III avatrombopag trial, 3 of 32 avatrombopag-treated patients experienced thromboembolic events during the core study [32]. Across the core and extension phases, events occurred at platelet counts ranging from 39-271 x 10^9^/L, and 3 of the 4 affected patients had multiple underlying thrombotic risk factors [32]. Conversely, in the L-PLUS 2 trial of lusutrombopag in CLD, two thrombotic events occurred among lusutrombopag-treated patients and two among placebo-treated patients [33]. These findings illustrate the difficulty of attributing thrombosis solely to TPO-RA exposure in populations with substantial underlying thrombotic risk.

Meta-analyses have produced differing estimates of the magnitude of this association. Tjepkema et al. reported a numerical but statistically non-significant increase in thrombosis with TPO-RAs (RR 1.82; 95% CI, 0.78-4.24), while Shen et al. similarly found no statistically significant increase across randomized controlled trials (OR 1.76; 95% CI, 0.78-4.00) [14,34]. Dong et al. also found no statistically significant increase in overall thrombosis across pooled randomized trials, although reported thrombotic event rates were substantially higher in studies involving treatment for more than six months compared with shorter exposure (4.7% versus 0.1%) [27]. More recently, however, Dai et al. reported thromboembolic events in 0.72% of TPO-RA-treated patients compared with 0.23% of controls across randomized trials (Peto OR 3.25; 95% CI, 1.11-9.51), while prospective evidence suggested that arterial thrombotic risk became more prominent with prolonged treatment [35].

Real-world evidence further supports the importance of thrombotic risk during TPO-RA therapy. Goncalves et al. identified 37 thrombotic events among 26 adults with ITP over 1,365 person-years, corresponding to an incidence of 2.71 events per 100 person-years, and TPO-RA therapy was associated with thrombosis in their analysis [26]. Most recently, Zordan et al. reported a one-year weighted cumulative incidence of thrombosis of 5.1% among patients receiving eltrombopag compared with 1.9% among those receiving romiplostim, with an adjusted hazard ratio of 1.93 (95% CI, 1.11-3.34) [13]. Although this finding raises the possibility of differences between individual TPO-RAs, the observational nature of the study prevents the conclusion that eltrombopag is intrinsically more thrombogenic than romiplostim [13].

Overall, current evidence supports a persistent but heterogeneous thrombotic association with TPO-RA therapy [13,14,26,27,34,35]. Thrombosis does not appear to depend solely on treatment-induced thrombocytosis, and several analyses have reported higher thrombotic event rates with longer treatment exposure [27,30,35]. However, randomized studies have not consistently demonstrated a statistically significant increase in thrombosis, and comparative differences among individual TPO-RAs remain uncertain [13,14,34]. The available evidence therefore favors individualized thrombotic risk assessment rather than reliance on platelet count alone when evaluating patients receiving TPO-RA therapy.

Methodology of TPO mimetic dosing and administration modifications to mitigate thrombosis risk

TPO-RA dosing strategies aim to achieve a platelet count sufficient to reduce bleeding rather than normalize it. In clinical studies of romiplostim, doses were adjusted to maintain platelet counts within a target range of 50-200 x 10^9^/L, supporting the principle of maintaining the lowest platelet count needed to stop bleeding [36]. Current dosing recommendations for romiplostim, eltrombopag, and avatrombopag similarly use the lowest effective dose necessary to maintain a platelet count of greater than or equal to 50 x 10^9^/L [21,37,38]. Dose reduction or temporary interruption is used to limit excessive platelet responses. Romiplostim dosing is reduced when platelet counts remain above 200-400 x 10^9^/L for two consecutive weeks and withheld above 400 x 10^9^/L, with treatment resumed at a reduced dose after the platelet count falls below 200 x 10^9^/L [21]. Eltrombopag and avatrombopag similarly employ dose reductions at platelet counts of 200-400 x 10^9^/L and temporary interruption above 400 x 10^9^/L [37,38]. Platelets are monitored weekly during dose adjustments and monthly after a stable dose or platelet response has been achieved [21,37,38]. These algorithms limit excessive platelet elevations while preserving a hemostatic response; however, they have not been prospectively demonstrated to prevent TPO-RA-associated thrombosis. Importantly, since thrombotic events have not demonstrated a consistent relationship with platelet count, these approaches should not be interpreted as eliminating thrombotic risk [2,30,32,36].

Lusutrombopag dosing differs from these other therapies. It is given daily as a fixed 3 mg for seven days prior to a scheduled procedure in patients with CLD, rather than continuously titrated according to platelet response [24,33]. In L-PLUS 2, thrombotic events were detected prospectively in both lusutrombopag- and placebo-treated patients, further demonstrating the importance of considering underlying thrombotic risk rather than platelet response alone [33].

Dose modification should therefore be accompanied by assessment of patient-specific thrombotic risk. Ghanima et al. recommended considering previous thromboembolism, splenectomy, antiphospholipid antibodies, and concomitant prothrombotic medications when initiating TPO-RA therapy and correcting modifiable risk factors when possible [2]. The authors also recommend thromboprophylaxis in appropriate surgical settings when a safe platelet count has been achieved and suggest that antiplatelet therapy or anticoagulation may be considered in selected patients at high thrombotic risk once platelet counts exceed 50 x 10^9^/L [2]. These recommendations represent individualized risk-management strategies rather than general prophylactic antithrombotic therapies to prevent TPO-RA-associated thrombosis.

Management after a thrombotic event remains less well defined. The literature does not establish a standardized strategy for temporary interruption, permanent discontinuation, switching between TPO-RAs, or rechallenge following thrombosis. This is particularly relevant because platelet counts generally return toward pretreatment levels following TPO-RA discontinuation, and transient rebound thrombocytopenia below baseline has been reported in some patients [2]. Decisions regarding continued TPO-RA therapy after thrombosis should therefore be individualized according to the thrombotic event, platelet count, bleeding risk, need for anti-thrombotic therapy, underlying thrombotic risk factors, and continued need for platelet support. At present, there is insufficient prospective evidence to recommend a standardized strategy for restarting TPO-RAs following thrombosis.

Overall, available evidence supports maintaining the lowest effective TPO-RA dose necessary to achieve a hemostatic platelet count, modifying or withholding treatment in response to excessive platelet elevations, and reassessing patient-specific thrombotic risk throughout therapy [2,36]. These measures should be regarded as rational risk-mitigation strategies rather than interventions proven to prevent thrombosis. The absence of standardized evidence regarding discontinuation and rechallenge following a thrombotic event remains an important gap in the literature.

Discussion

TPO-RAs have substantially expanded therapeutic options for patients with thrombocytopenic disorders, particularly ITP, by providing an effective means of increasing platelet production and reducing bleeding risk. However, the evidence reviewed here demonstrates an important clinical paradox: therapies intended to correct thrombocytopenia are associated with a thrombotic safety signal in a population that may already possess an elevated baseline risk of thrombosis. The magnitude of this association remains uncertain. Earlier studies generally demonstrated increased thrombotic events without statistical significance, whereas more recent evidence has strengthened the association [14,27,34,35]. Dong et al. found a non-significant increase in thrombotic events with thrombopoietic agents, whereas Dai et al. subsequently identified a statistically significant increase in thromboembolic events across randomized trials [27,35]. These differing results illustrate the challenge of characterizing an uncommon adverse event in trials primarily designed to evaluate platelet response and bleeding outcomes.

Thrombotic events have been observed in romiplostim, eltrombopag, avatrombopag, and lusutrombopag despite differences in molecular structure, receptor-binding site, route of administration, or treatment duration [30-33]. While thrombotic risk should be identified as a safety concern associated with the TPO-RA drug class, evidence does not establish equivalent risk among the agents. The 2026 FAITH target-trial emulation reported a higher one-year weighted cumulative incidence of thrombosis with eltrombopag than romiplostim, with an adjusted hazard ratio of 1.93 [13]. However, no significant difference was observed during the first six months, with differences emerging later from relatively few events [13]. Despite target-trial emulation and statistical adjustment, the observational design cannot exclude residual confounders. These findings raise the possibility of different thrombotic risks between agents but do not conclude that eltrombopag is intrinsically more thrombogenic than romiplostim [13]. Current evidence supports a class-associated thrombotic safety signal but remains insufficient to define a reliable hierarchy of risk among individual TPO-RAs.

The relationship between platelet count and thrombosis is similarly complex. Although thrombocytosis represents an intuitive mechanism through which TPO-RAs could promote thrombosis, clinical evidence does not demonstrate a consistent platelet count-dependent relationship. Long-term romiplostim data did not demonstrate an association between platelet count and thrombotic events, while thromboembolic events in the avatrombopag trial occurred across platelet counts ranging from 39 to 271 × 10^9^/L [30,32]. Ghanima et al. similarly reported that thromboembolic events were not consistently associated with thrombocytosis or higher TPO-RA doses and that a substantial proportion occurred at platelet counts below the normal range [2]. These observations suggest that maintaining platelet counts below an arbitrary upper threshold cannot serve as the sole strategy for mitigating thrombotic risk. Current recommendations to use the lowest effective TPO-RA dose necessary to achieve a hemostatic platelet count remain appropriate but should be viewed as a strategy to avoid unnecessary platelet elevation rather than an intervention to prevent thrombosis.

Patient characteristics may therefore be at least as important as the achieved platelet count when considering thrombotic risk. ITP itself is associated with thrombotic complications despite its characteristic thrombocytopenia [26]. Older age, previous thrombosis, splenectomy, antiphospholipid antibodies, and concomitant prothrombotic medications should be considered when assessing thrombotic risk in patients receiving TPO-RAs [2,26]. Treatment duration may also be relevant, as Dong et al. reported higher thrombotic event rates in studies involving treatment for more than six months, while Dai et al. reported increasing arterial thrombotic event rates with longer prospective exposure [27,35]. These findings do not establish treatment duration as an independent causal determinant of thrombosis, but they support continued reassessment of thrombotic risk throughout therapy rather than assessment only at treatment initiation.

These observations have important implications for clinical monitoring. Current TPO-RA treatment strategies appropriately emphasize platelet-guided dose titration, use of the lowest effective dose, and reduction or interruption following excessive platelet responses [2,36,21,37,38]. Nevertheless, the evidence reviewed here indicates that platelet-count monitoring alone cannot fully characterize thrombotic risk. Patient-specific risk factors should therefore be reassessed throughout treatment, particularly when new prothrombotic conditions or exposures develop [2]. At present, the literature reviewed does not provide evidence supporting routine radiographic screening for asymptomatic thrombosis in all patients receiving TPO-RAs. Future studies should determine whether specific high-risk populations could benefit from additional surveillance beyond routine clinical and platelet assessment.

Management after adverse thrombus formation remains another major evidence gap, as there is not a standardized approach to temporary TPO-RA interruption, permanent discontinuation, switching between agents, or subsequent rechallenge. Discontinuation can also be followed by a return of platelet counts toward pre-treatment levels and, in a minority of patients, transient thrombocytopenia below baseline [2]. Consequently, management following thrombosis must balance the severity and location of the thrombotic event against platelet count, bleeding risk, need for antithrombotic therapy, underlying thrombotic risk, and the continuing indication for TPO-RA treatment. Prospective studies evaluating continuation, interruption, switching, and rechallenge following thrombosis are needed before standardized recommendations can be developed.

Several limitations of the existing evidence should be acknowledged. Thrombotic events are relatively uncommon, leaving many randomized trials underpowered to detect clinically meaningful differences and resulting in wide confidence intervals in several pooled analyses [14,27,34]. Considerable heterogeneity also exists with respect to TPO-RA agent, treatment duration, underlying diagnosis, baseline thrombotic risk, and definitions of thrombotic outcomes. Furthermore, much of the available evidence concerns adults with ITP, limiting the extrapolation of data and generalizability to pediatric patients and other indications, such as aplastic anemia, MDS, and CLD. Observational cohorts offer longer follow-up and greater representation of the patients encountered in clinical practice but remain susceptible to residual confounding and treatment-selection bias. This limitation is particularly relevant when interpreting comparative observational studies such as FAITH [13]. Finally, prospective evidence demonstrating that currently employed dose-modification or monitoring strategies to reduce thrombotic events remains limited.

Taken together, the available evidence supports moving beyond a solely platelet-centered approach toward a more individualized model of thrombotic risk assessment during TPO-RA therapy. Avoidance of excessive platelet counts remains appropriate, but thrombotic risk appears to reflect an interaction among the underlying disease, patient-specific risk factors, treatment exposure, and TPO-RA therapy rather than platelet count alone [2,26,27]. Current evidence does not justify selecting one TPO-RA over another solely based on presumed thrombotic safety, although emerging comparative data warrant further investigation [13]. Future prospective studies should incorporate standardized reporting of arterial and venous thrombotic outcomes, stratification by baseline risk and treatment duration, direct comparisons among individual TPO-RAs, and systematic evaluation of treatment modification and rechallenge after thrombosis. Such evidence could allow future guidance to incorporate individualized thrombotic risk more explicitly while preserving the established therapeutic benefits of TPO-RA therapy.

## Conclusions

TPO-RAs have proven to be beneficial in various clinical scenarios requiring restoration of platelet counts to reduce bleeding risk and manage hematologic disorders. When prescribing these drugs that are meant to promote thrombocytosis, it is important to consider the associated thrombotic risks, which appear to be multifactorial. Discussion of the literature emphasizes the need for individualized thrombotic risk assessment and continued monitoring during TPO-RA therapy that accounts for patient-specific risk factors, such as prior thrombosis, advanced age, splenectomy, antiphospholipid antibodies, or underlying ITP. Higher platelet counts have not consistently been associated with increased thromboembolic events; thus, dose adjustments are not sufficient to modify thrombotic risk. Evidence also does not show one drug to have a mechanism of action that is more prothrombotic. Going forward, clinicians using TPO-RAs should be aware of potential thrombotic risks and consider individual predisposing factors when prescribing.
